# The effect of hydroxyzine on treating bruxism of 2- to 14-year-old children admitted to the clinic of Bandar Abbas Children Hospital in 2013-2014

**Published:** 2015

**Authors:** M Rahmati, A Moayedi, S Zakery Shahvari, J Golmirzaei, M Zahirinea, B Abbasi

**Affiliations:** * Department of Infectious Diseases, Children’s Clinical Research Development Center, Hormozgan University of Medical Sciences, Bandar Abbas, Iran; ** Pediatric Neurology, Hormozgan University of Medical Sciences, Bandar Abbas, Iran; *** Department of Pediatrics, Hormozgan University of Medical Sciences, Bandar Abbas, Iran; **** Pediatric Neurology Research Center, Shahid Beheshti University of Medical Sciences, Tehran, Iran; ***** Hormozgan University of Medical Sciences, Bandar Abbas, Iran

**Keywords:** bruxism, hydroxyzine, children

## Abstract

**Introduction.** Bruxism is to press or grind teeth against each other in non-physiologic cases, when an individual does not swallow or chew. If not treated, teeth problems, stress, mental disorders, frequent night waking, and headache is expected. This research aimed to study the effect of hydroxyzine on treating bruxism of 2- to 14-year-old children admitted to the clinic of Bandar Abbas Children Hospital.

**Methodology.** In this clinical trial, 143 children with the ages between 4-12 years were admitted to the Children Hospital and were divided randomly into test and control groups. The test group consisted of 88 hydroxyzine-treated children and the control group consisted of 55 children who used hot towels. Both groups were examined in some stages including the pre-test stages or the stage before starting treatments at two, four, and six weeks and four months after stopping the treatment. The effects of each treatment on reducing bruxism symptoms were assessed by a questionnaire. The data were analyzed by using SPSS in descriptive statistics, t-test, and ANOVA.

**Results.** As far as bruxism severity was concerned, the results showed a significant difference between the test group members who received hydroxyzine and the control group members who received no medication. T-test results showed a statistically significant difference between the test and the control groups in the second post-test (four weeks later) (p. value ≤ 0.05). Mean of the scores of bruxism severity in the test group has changed significantly in the post-test (at two weeks, four weeks, and six weeks later) as compared to the pre-test. Whereas, as far as the response to the treatment, no significant difference was recorded between the control group and the test group 4 weeks after the treatment.

**Discussion.** The results showed that prescribing hydroxyzine for 4 weeks had a considerable effect in diminishing bruxism severity between the test groups.

## Introduction

Bruxism is to press or grind teeth against each other in non-physiologic cases, when an individual does not swallow or chew [**1**]. Bruxism may occur during days and nights and its prevalence among children varies between 7 to 88 percent [**2**]. Its severities among 4- to 6-year-old and 7- to 10-year-old children were reported to occur in 55% cases, and in 35%, respectively [**3**]. However, bruxism causes remain unknown [**4**,**1**]. It seems that the involvement of central nervous system and/ or the autonomic nervous system in bruxism exceeds the one of environmental factors. Further, the factors of peripheral sensory may contribute in bruxism by interfering with the mechanisms of sleep and wakefulness [**5**]. Some studies reported several causes including stress, local factors - such as dental problems, systemic factors - such as allergies and intestinal parasites [**6**-**8**]. Other studies stated that biological factors such as family, and nervous-psychological factors are effective in bruxism; but the absence of no factor has not been proven yet [**9**]. The pathogenesis of the illness is unknown and there are two hypotheses for this habit: the first hypothesis includes local diseases of environmental morphology such as malocclusion and, the other factor encompasses central disorders in basal ganglia region, which is a striatum dopaminergic activity. In a case report from 2005, Chen and Loo discovered a reduction of the frontal lobe hypoperfusion and a lack of sensitivity to L-dopa or bromocriptine and found a suitable response to metoclopramide in three patients with daytime and nighttime bruxism. The pre-synaptic sensitivity of dopamine receptors determined the relationship between bruxism and both hypo and hyperdopaminergic statuses [**8**]. However, no effective treatment has been found for bruxism habit to cure it permanently [**1**]. Therefore, the remedial measurements are based mostly on palliative treatments and on the reduction of the pathological complications in the masseter structure [**5**]. If not treated, teeth problems, stress, mental disorders, frequent night waking and headache can be expected [**10**]. Solving the factors that may reduce anxiety improves considerably as well as removes the children’s fears effectively. In resistant cases, one should refer to a dentist to reduce the dental disorder process and remove muscle and joint pains of temporomandibular joint [**11**]. Contradictory effects and side effects such as drowsiness and dependence have requested a limited use of medication, especially Propranolol, Bromocriptine, Clonazepam, Botulinum toxin, Fluoxetine or Benzodiazepines in the treatment [**12**-**14**]. Hydroxyzine is the first generation of strong antagonist of H1 receptors. It has both anti-histaminergic effects and anti-dopaminergic effects. The hydroxyzine’s possible effectiveness mechanism in the bruxism treatment can be attributed to the pharmaco-pathological relationship between the anti-dopaminergic activity of hydroxyzine and the disorder in the dopaminergic center of the brain [**13**]. In this case study, we conducted a comparative examination on the hydroxyzine treatment and hot towels in 4- to 12-year-old children with bruxism admitted to the Children Hospital in order to prevent the complications of the disease, suggesting a suitable treatment method. 

## Material and Methods

This study was a clinical trial on the children with bruxism admitted to the Children Hospital of Bandar Abbas from autumn 2013 to summer 2014. A consensus sampling method was employed. In addition, 143 children with the ages between 2 and 14 years were admitted to the hospital complaining of bruxism. The patients were divided into a control group (55 children) who only used hot towels for the treatment and the test group (88 children) who were treated with both hot towels and hydroxyzine (5 mg every night and 10 mg from the fifth night on for the same month). At the beginning of the study, questionnaire No. 1 and questionnaires No. 2, 3, 4, 5 on bruxism complaint were prepared every fortnight for up to six weeks. All the questionnaires measured bruxism severity based on standard. Questionnaire No. 5 was prepared four months after stopping the treatment in order to examine symptoms, recurrence, and drug effect. The questionnaire’s items consisted of an observance of bruxism or pressing teeth during night or day and/ or during night and day, masseter muscle fatigue one hour after waking and/ or continuous masseter muscle fatigue for all day, application of cords (dental facets) at night, headache for one hour after waking, headache for all day, change of teeth or their erosion, morning fatigue, daily sleepiness, neck pain as well as clinical information such as age and gender. A positive reply to each question had a score. Based on the acquired scores, the patients were divided into three groups: mild, moderate, and severe with the scores between 1 (minimum symptoms) to 12 (maximum severity of symptoms). The children whose parents were unwilling to cooperate or were sensitive to hydroxyzine, with seizure disorder or tic disorder and those who consumed anti-anxiety medications, with sleep disorders such as obstructive sleep apnea, and mentally retarded children, were excluded from the study. The collected data were coded and entered in SPSS. T-test was used for the data analysis and the demographic data were reported in tables and diagrams. 

## Results

The males admitted children complaining of bruxism exceeded the females. The maximum and minimum frequencies belonged to the age group of 6-8 years old (31.8%) and the group of over 10 years old (2.3%), respectively. The mean ages of the participants in the test group and the control group were 6.26 and 6.32, respectively. More than 82% of the patients admitted in the hospital complaining of bruxism were in the age group of lower than 8 years old. In the test group, bruxism was the most frequently reported in the pretest stage, with a severity of 4-6. In the first stage, two weeks after starting treatment, the severity of symptoms was between 3 and 5. In the second stage, four weeks after treatment, the symptoms’ severity was between 0 and 2. In the final stage of the post-test, six weeks after treatment, the symptoms’ severity was between 0 and 2. The results showed that bruxism severity can be reduced considerably by using a four-week treatment with hydroxyzine and hot towels. In contrast, in the control group treated with hot towels in the pre-treatment stage, the maximum frequency of bruxism severity was between 4 and 6. In the first stage, two weeks after treatment, the severity was between 3 and 6, in the second stage, four weeks after the treatment, the severity was between 0 and 2. Six weeks after the treatment, it was between 2 and 4. The data showed that a four-week treatment might reduce bruxism severity. Four months after stopping the treatment, the mean of bruxism severity in the group was 3.75. The means of bruxism severity in the test and the control groups were 5.07 and 4.91, respectively. However, six weeks later, bruxism severity was reduced in the test and the control groups, being of 0.68 and 2.73, respectively. Therefore, the mean scores of bruxism severity were considerably reduced six weeks after hydroxyzine consumption. Four months after stopping the treatment, the mean score of the symptoms’ severity in the group treated with hydroxyzine and hot towels was 3.73. The statistical test results showed bruxism symptoms recurrence four months after stopping the treatment in both groups. The mean scores of bruxism severity in the test group in the post-tests, at two weeks, four weeks, and six weeks after treatment, was significantly changed as compared to the pre-test. However, no considerable changes were observed in the mean scores of the control group in 4 stages. 

No statistically significant difference was recorded in the two study gender groups as far as the mean score of bruxism severity was concerned (P.V = 0.05). Based on the ANOVA results, the mean score of bruxism severity among the age groups in the test group (treatment with hydroxyzine) did not differ significantly. The difference was only significant in the fourth week after the treatment started (merely between the age groups of 2-4 and 6-8 years old).

The figure shows that bruxism severity was reduced in both methods. 

**Fig. 1:**
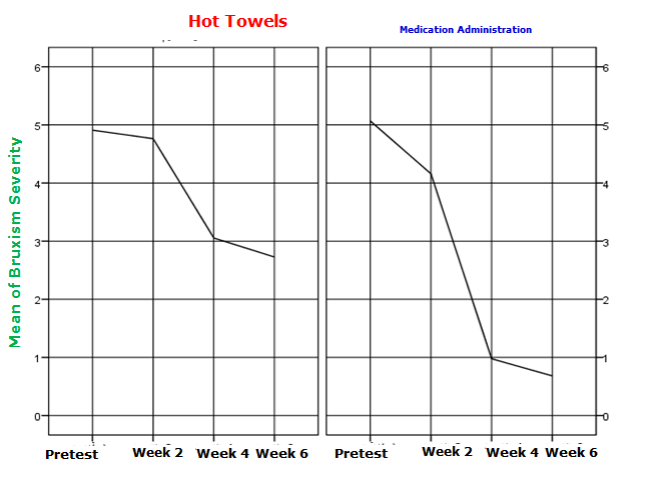
Reduced bruxism severity in both methods

## Discussion and conclusion

According to the research results, the effectiveness of hydroxyzine in treating bruxism exceeded the one of hot towel. The result was also reported in the report-case study of Ghanizadeh et al., 2013 on three individuals treated with hydroxyzine [**12**]. Our study is the first that examined the effect of hydroxyzine on different genders and ages of children. It showed the effect of hydroxyzine on males, which exceeded, being more effective after 4 weeks of treatment in the age groups of 2-4 and 6-8 years old. The study’s strong point was the follow up of the patients 4 months after stopping the treatment in order to examine the effect of the drug on long term after its stopping and its recurrence of symptoms. The results showed that the patients encountered symptoms recurrence, which indicated that the drug had an important role in the symptomatic treatment of the disease. Ghanizadeh mentioned that taking advantage of an anti dopaminergic effect was the base of the hydroxyzine effectiveness [**12**]. Cook et al. also mentioned the anti-dopaminergic effect of hydroxyzine in treating bruxism [**13**]. Our study had some limits as for example gaining the data through the parents’ reports in which most of the mild or moderate cases might not have been reported due to the fact that both parents and children were sleeping at the same time. Therefore, other methods for bruxism diagnosis should be taken into consideration. Another limit was to report cases qualitatively based on the parents’ opinions. Another limit was the allocation of a long time for following up the patients as it might lead to missing out some participants [**15**,**16**]. As psychological theory suggests bruxism is the symbol of a personality disorder or stress intensification [**10**] and is common among mentally retarded children suffering from musculoskeletal disorders (cerebral palsy). Further, researchers should consider systemic etiologic factors such as intestinal parasites, sub-clinical nutritional deficiencies, and allergies [**17**]. Short- and long-term side effects of the treatment with hydroxyzine should also be taken into consideration in order to make suitable decisions on treating patients.

## Conclusion

The results showed that the intensity of bruxism symptoms in the control and the test groups was reduced. However, this outcome was acceptable in the test group (who received hydroxyzine). 
